# Barriers to Universal Availability of Medications for Opioid Use Disorder in US Jails

**DOI:** 10.1001/jamanetworkopen.2025.5340

**Published:** 2025-04-16

**Authors:** Elizabeth Flanagan Balawajder, Lori Ducharme, Bruce G. Taylor, Phoebe A. Lamuda, Marynia Kolak, Peter D. Friedmann, Harold A. Pollack, John A. Schneider

**Affiliations:** 1 Public Health Department, NORC at the University of Chicago, Chicago, Illinois; 2 National Institute on Drug Abuse, National Institutes of Health, Bethesda, Maryland; 3 Department of Geography and Geographic Information Science, University of Illinois at Urbana-Champaign, Urbana; 4 Office of Research, Baystate Health and University of Massachusetts Chan Medical School-Baystate, Springfield; 5 Crown Family School of Social Work, Policy and Practice, University of Chicago, Chicago, Illinois; 6 Department of Public Health Sciences, University of Chicago, Chicago, Illinois; 7 Department of Medicine, University of Chicago, Chicago, Illinois

## Abstract

**Importance:**

Many of the approximately 2 million people being held in US correctional facilities are experiencing an opioid use disorder (OUD). Providing medications for OUD (MOUD) to this population is, therefore, essential to curb the opioid crisis.

**Objective:**

To examine the types of MOUD jails are making available, factors associated with availability, and additional supports needed for jails to address implementation challenges.

**Design, Setting, and Participants:**

This survey study used a cross-sectional survey of jails conducted between February 2 and July 1, 2023, to explore how they administer MOUD. Publicly available county-level data were connected with the survey responses to assess how variables in the surrounding community were associated with MOUD availability. The survey was administered to jails via mail, telephone, and online survey link. Participants included jails with MOUD available that completed the survey.

**Exposures:**

Urbanization, average daily population, availability of a health care professional to administer MOUD, whether the state expanded Medicaid, average drive time to MOUD in the county, county overdose rate, and county social vulnerability were assessed.

**Main Outcomes and Measures:**

The primary outcome was the type of MOUD available in the jail, including buprenorphine, methadone, or naltrexone, or all 3 medications. Binary logistic regressions were conducted to identify the characteristics of jails and county-level factors associated with offering the medications.

**Results:**

A total of 462 jails were invited to complete the survey based on responses to a previous nationally representative survey of jails, in which they indicated that MOUD was available to individuals in their facility. A total of 265 US jails with MOUD available were included in the analysis, representative of 1243 jails nationwide with MOUD available after weighting (812 jails [65.3%] provided buprenorphine, 646 jails [52.0%] provided naltrexone, 560 jails [45.0%] provided methadone, and 343 jails [27.6%] provided all 3 medications). Availability was associated with urbanicity, location in a Medicaid expansion state, county opioid overdose rate, and county social vulnerability. Common challenges included jail policies and procedures and the logistical accessibility of the medication.

**Conclusions and Relevance:**

The findings of this survey study of US jails demonstrate that jails with MOUD available still experience challenges with making all 3 types of medication available to anyone held within their facility. Policy, regulatory, financing, staffing, and educational solutions are needed to ensure that all detainees with OUD have access to treatment while incarcerated.

## Key Points

**Question:**

How are medications for opioid use disorder (MOUD) provided, and what additional supports are needed among jails with MOUD available?

**Findings:**

In this survey study of 265 jails with MOUD available, buprenorphine was the most common medication (65.3%), and 27.6% made all 3 types of MOUD (buprenorphine, naltrexone, and methadone) available.

**Meaning:**

These findings suggest that even within jails that have MOUD, there are barriers to making all 3 types of medication available to everyone, resulting in many people not receiving recommended care while detained; policy, regulatory, financing, staffing, and educational solutions are needed to address this ongoing crisis.

## Teaser

This survey study examines the types of medications for opioid use disorder available in US jails, factors associated with availability, and additional supports needed for jails to address implementation challenges.

## Introduction

At any given time in the US, approximately 2 million people are held in correctional facilities,^1^ and some research estimates that close to one-third of the US population will be arrested by early adulthood,^2^ suggesting that a high proportion of US individuals come into contact with correctional facilities at some point in their lives. The 2019 Census of Jails found that 15% of the jail population screened positive for an opioid use disorder (OUD),^3^ compared with approximately 3% estimated in the general population.^4^ Even with recent decreases in overdose rates, more than 64 000 individuals in the US died of overdose in the year ending in August 2024.^5^ Treating OUD among people impacted by criminal legal systems thus provides a critical opportunity for curbing the overdose epidemic.

Medication for OUD (MOUD) is standard of care for OUD. In the general population, MOUD has been shown to reduce illicit opioid use, opioid-related hospitalizations, and overdose, even when compared with inpatient care for OUD.^6,7,8^ MOUD includes treatment with buprenorphine, methadone, or naltrexone. MOUD provision in jail settings has been shown to decrease the risk of overdose upon release into the community,^9,10,11^ and a recent study^12^ estimated that 668 lives would be saved per 10 000 incarcerated persons if every detainee with an OUD was offered MOUD. Guidance from the American Society of Addiction Medicine^13^ states that all 3 medications should be made available to individuals with OUD in correctional settings. The medications have varying mechanisms of action and routes of administration and cost, making each type of MOUD suitable for different patients on the basis of their state of illness, treatment history, and preferences.^14,15,16,17,18^ Local availability after release also varies among medications, further emphasizing the importance of having the 3 options.^17,19^ Yet, previous research has found limited availability of MOUD in jails; less than one-half of jails nationwide provide this treatment, with higher proportions of MOUD jail deserts in counties with higher overdose rates.^3,20,21^ Recognizing jails and other correctional settings as a touchpoint for individuals with an OUD, recent policies and guidance have aimed to help increase the availability of MOUD in these settings.^22^ For example, the Medicaid Reentry Section 1115 Demonstration Opportunity offers states the ability to provide substance use treatment to individuals prior to their release from jail.^23^

Even within jails that offer MOUD, such treatment is typically not universally available. Instead, MOUD may be provided only to pregnant people or those already taking MOUD when entering the facility. Barriers to universal provision of MOUD within jails include perceived risk of diversion (ie, sale or trade of MOUD for unauthorized use), local jail policies, space limitations, short jail stays, limited staff, cost, and limited MOUD resources in the community to continue treatment on release.^24,25^ Despite recent updates to federal regulations for opioid treatment programs (OTPs),^26^ regulatory barriers continue to impede dispensing of methadone outside of OTPs, including in jails. Myriad policy, financing, and training solutions are likely needed to achieve broad implementation and delivery of comprehensive MOUD in jail settings. Better understanding of the current capacity and experience of jails is needed to inform next steps. This analysis builds upon a recent National Survey of Substance Use Services in US Jails^27^ to explore (1) how and to whom MOUD is provided in jails that do offer it; (2) what jail-level or external factors are associated with providing all 3 types of MOUD as recommended by American Society of Addiction Medicine guidance; and (3) what additional supports are needed for jails to provide these lifesaving medications.

## Methods

Findings for this study are based on a cross-sectional survey of US jails conducted between February 2 and July 1, 2023. This study received a determination as not human participants research from the institutional review board at NORC at the University of Chicago. Surveys sent to jails explained confidentiality and data protections. The study followed the American Association for Public Opinion Research (AAPOR) reporting guideline for surveys.

### Sample

These analyses include jails that provide MOUD. Jails were identified through a previous, nationally representative survey of 1028 jails conducted between June 2022 and April 2023, as described in Flanagan Balawajder et al^27^ (referred to as part 1). In part 1, all jails were asked whether MOUD had been available within the last 12 months to treat OUD. Jails indicating availability of any MOUD (462 jails) were eligible to participate in the follow-on survey (ie, part 2) to describe their MOUD services in detail. The part 2 survey response rate was 60.2% (278 of 462 jails).

### Data Collection

All 462 jails that met eligibility criteria were invited to complete the part 2 survey, administered via web, mail (paper), and telephone. Jails received an invitation email (if an email was available), postcard, up to 2 paper surveys in the mail, and multiple email reminders.

### Measures

The approximately 40-minute survey consisted of 75 items. The survey assessed the types of MOUD available in the jail, how and to whom it is provided, challenges and facilitators to providing it, and resources needed to expand MOUD availability.

#### Type of MOUD and To Whom It Is Available

Jails were asked, “Which types of medications (MAT) does your facility offer to detainees for the maintenance treatment of opioid use disorder?” with options including buprenorphine, methadone, naltrexone, none of the above, or don’t know. (The term *MAT*, or medication-assisted treatment, was used because it is most familiar to jail staff.) Respondents were asked to whom medications were made available, with response options including anyone with an OUD, anyone already taking a prescribed medication at the time of incarceration, pregnant people, people within several months of release, and certain exceptional cases. For each medication used, the survey ascertained how it was used (eg, continuity of care or prerelease induction) and provided (eg, jail’s own medical staff administers or contracted professional comes to jail).

#### Jail Size and Availability of Staff

MOUD implementation likely reflects jails’ capacity and resources. Thus, measures included a jail’s average daily population, and whether a health care professional was available to administer MOUD to individuals in the jail (yes or no).

#### County Variables

Because MOUD implementation may be influenced by jails’ outer context, we used county-level data available through the Opioid Environment Policy Scan (OEPS) database.^28^ We used 5 categories to define urbanization based on the National Center for Health Statistics’ Rural-Urban Classification Scheme^29^: large metropolitan counties (population ≥1 million), medium metropolitan counties (population 250 000-999 999), small metropolitan counties (population ≤250 000), micropolitan counties (have an urban cluster population ≥10 000), and noncore counties that do not have an urban cluster of at least 10 000 people. We assigned a value of 1 to all jails located in a state that expanded Medicaid prior to 2022.

Local availability of MOUD was assessed through mean drive times to the nearest MOUD treatment location in the county. The OEPS database calculates mean drive times for buprenorphine, methadone, and naltrexone using the Substance Abuse and Mental Health Services Administration treatment locator, and we used the lowest mean drive time to one of these treatment locations. The OEPS used 2020 data from the Centers for Disease Control and Prevention’s National Center for Health Statistics to compute the mortality rate from opioid overdose per 100 000 persons in the surrounding county.^28^

We used Centers for Disease Control and Prevention’s 2020 Social Vulnerability Index summary rank to assess county socioeconomic factors. This measure ranks counties according to factors such as poverty level, housing, and transportation.^30^ Our analysis treated social vulnerability as a continuous variable, with higher values reflecting greater vulnerability, allowing for a more granular assessment of how varying degrees of social vulnerability correlate with MOUD availability.

#### Challenges, Facilitators, and Resource, Training, and Technical Assistance Needs

All jails were asked to indicate their top 3 challenges with administering buprenorphine and methadone. Owing to similarities between response options, we collapsed the 18 response options into 5 categories: (1) policies and procedures (ie, concerns about Drug Enforcement Administration regulations; state, county, or jail policies and regulations; concerns about security and diversion; policies restricting who is able to receive medication; funding or cost; and jail is not a licensed OTP); (2) logistical accessibility of medication (ie, transportation and storage, limited authorized prescribers to administer, limited staff to transport patients to receive a dose, or short duration of stays); (3) staff knowledge and attitudes (ie, limited knowledge among staff about the medication, practitioner attitudes about the medication, or jail staff believe in a 12-step or abstinence-based approach); (4) patient knowledge and attitudes (ie, patients ask for another type of medication, attitudes about the medication, and adverse effects); and (5) community availability (ie, lack of linkages to community-based MOUD providers or medication is difficult to access in the community and do not want to start someone on a medication they are unable to continue). In addition, jails were asked 3 multiple choice questions to indicate what technical assistance (ie, screening tools, clinical practice guidelines, identifying best MAT for patient, staff education, counseling, community resources, or other), training (ie, buprenorphine waivers, partnering with OTPs, memoranda of understanding to support reentry, community linkage partnerships, administration of medication, injectable MOUD, and activating or reactivating Medicaid), and resources (ie, medical staff, same-day MOUD access, addressing stigma, information on MOUD and pregnancy, MOUD training for clinical staff, information for becoming an OTP, and MOUD technical assistance) they would most benefit from.

### Statistical Analysis

All analyses were conducted using SPSS statistical software version 29.0 (IBM). Data were weighted to be representative of over 1300 jails nationwide offering MOUD (based on results from the part 1 survey) and to account for jail-level nonresponse. We produced descriptive statistics of all measures for our sample to assess types of MOUD available. We conducted 4 binary logistic regressions to identify the characteristics of jails and county-level factors associated with offering (1) all 3 medications, (2) buprenorphine, (3) methadone, and (4) naltrexone. These outcome variables were not mutually exclusive and do not imply universal availability. Independent variables were selected for inclusion based on previous research^27^ and application of the Consolidated Framework for Implementation Research, a well-established implementation science conceptual framework that considers key areas of influence internal and external to an organization.^31^ As suggested by the framework, each variable included in the model aligns with either the inner setting (ie, organizational size and staff availability) or the outer context in which those organizations operate (ie, urbanicity, drive time, overdose rate, and social vulnerability).^31^ Finally, we produced descriptive statistics to assess challenges, facilitators, and resource needs among jails to promote broader uptake of MOUD. Only jails with complete data for all variables of interest were included in the analyses. Statistical significance was defined as 2-sided *P* < .05.

## Results

Of the 278 responding jails, 265 (57.4%) provided complete data for all variables of interest in the regression; these were weighted to represent 1243 jails across the US estimated to be providing some form of MOUD to at least some individuals. We compared respondents with nonrespondents and found no differences based on jail size (χ^2^ = 1.58; *P* = .81), region (χ^2^ = 1.91; *P* = .59), or urbanicity (χ^2^ = 4.50; *P* = .35). As shown in Table 1, approximately one-quarter (343 jails [27.6%]) of jails with MOUD available indicated having all 3 medications (ie, buprenorphine, methadone, and naltrexone). Only 210 jails (16.9%) made all 3 types of MOUD available to anyone with an OUD. Of MOUD offered, buprenorphine was the most common (812 jails [65.3%]), followed by naltrexone (646 jails [52.0%]), and methadone (560 jails [45.0%]). MOUD was most commonly provided for continuing treatment that had started prior to an individual’s entry into the facility (656 jails [86.8%] for buprenorphine; 449 jails [87.4%] for methadone). Buprenorphine was about equally likely to be administered by in-house medical staff (331 jails [43.9%]) as by contracted medical staff (367 jails [48.6%]). Where methadone was available, approximately one-half of jails (258 jails [50.5%]) transported the medication to their jail, whereas almost one-third (159 jails [31.2%]) transported patients to a local OTP.

**Table 1.** Description of Sample and Available Types of MOUD Among Jails That Offer MOUD

Characteristics	Jails, No. (%)	
	Weighted (N = 1243)^a^	Unweighted (N = 265)
Mean daily jail population		
0-25	166 (13.4)	37 (14.0)
26-50	128 (10.3)	30 (11.3)
51-100	217 (17.5)	50 (18.9)
101-200	234 (18.8)	52 (19.6)
>200	498 (40.0)	96 (36.2)
Urbanization		
Large metropolitan	230 (18.5)	48 (18.1)
Medium metropolitan	226 (18.2)	46 (17.4)
Small metropolitan	191 (15.3)	40 (15.1)
Micropolitan	305 (24.6)	68 (25.7)
Noncore	291 (23.4)	63 (23.8)
Have staff available to administer MOUD	1110 (89.3)	237 (89.4)
State expanded Medicaid	933 (75.1)	212 (80.0)
Drive time to MOUD provider in county where jail is located, mean (SD), min	13.4 (12.3)	13.5 (12.6)
Overdose mortality rate in county where jail is located, deaths/100 000, mean (SD)	26.7 (13.4)	26.2 (12.8)
Social Vulnerability Index percentile rank, mean (SD)	0.47 (0.27)	0.46 (0.26)
Types of MOUD available and how they are used		
Buprenorphine	812 (65.3)	181 (68.3)
Use of buprenorphine		
Continuity of care or maintenance	659 (86.8)^b^	149 (87.1)
Initiation and continuation for pregnant people only	221 (29.1)^b^	51 (29.8)
Initiation and continuation for any detainees	404 (53.0)^b^	98 (57.3)
Detox	282 (37.0)^b^	64 (37.4)
Prerelease induction	256 (33.6)^b^	63 (36.8)
Managing pain	23 (3.1)^b^	6 (3.5)
Administration		
Contracted staff comes to jail to administer	367 (48.6)^b^	81 (47.9)
Jails own staff administers	331 (43.9)^b^	77 (45.6)
Detainees are sent out to licensed practitioner	53 (7.1)^b^	10 (5.9)
Methadone	560 (45.0)	123 (46.4)
Use of methadone		
Continuity of care or maintenance	449 (87.4)^c^	100 (87.7)
Initiation and continuation for pregnant people only	144 (28.1)^c^	32 (28.1)
Initiation and continuation for any detainees	121 (23.6)^c^	28 (24.6)
Detox	57 (11.0)^c^	12 (10.5)
Prerelease induction	42 (8.2)^c^	10 (8.8)
Managing pain	19 (3.8)^c^	4 (3.5)
Administration		
Jail transports medications from a local OTP to the jail	258 (50.5)^c^	59 (52.2)
Jail transports patients to a local OTP	159 (31.2)^c^	34 (30.1)
Jails operates own licensed OTP	38 (7.5)^c^	10 (8.8)
Other	172 (33.8)^c^	35 (31.0)
Naltrexone	646 (52.0)	143 (54.0)
Use of naltrexone		
Continuity of care or maintenance	377 (62.7)^d^	86 (64.2)
Initiation just prior to release	369 (61.5)^d^	84 (62.7)
Initiation and continuation of treatment during detainment	324 (53.9)^d^	74 (55.2)
Other	27 (4.5)^d^	6 (4.5)
To whom MOUD is available		
Anyone with an OUD	430 (43.7)^e^	101 (47.4)
Anyone who was already taking a prescribed medication at the time of incarceration	782 (79.7)^e^	172 (80.8)
Pregnant people receiving medication at time of incarceration	696 (70.9)^e^	151 (70.9)
People within several months of release	197 (20.0)^e^	46 (21.6)
Certain exceptional cases	81 (8.3)^e^	17 (8.0)
Offers all 3 types of MOUD to at least some individuals in their jail	343 (27.6)	79 (29.8)
Offers all 3 types of MOUD to anyone with an OUD	210 (16.9)	52 (19.6)

Abbreviations: MOUD, medications for opioid use disorder; OTP, opioid treatment program; OUD, opioid use disorder.

^a^ Data from 265 jails were weighted to be representative of 1243 jails nationwide.

^b^ Percentages refer to jails offering buprenorphine that completed this item (n = 762); those that provide MOUD to only pregnant people (n = 52 [4.0%]) were not included in this question.

^c^ Percentages refer to jails offering methadone that completed this item (n = 514); those that provide MOUD to only pregnant people (n = 52 [4.0%]) were not included in this question.

^d^ Percentages refer to jails offering naltrexone; those that provide MOUD to only pregnant people (n = 52 [4.0%]) were not included in this question.

^e^ Percentages refer to those who knew the type of MOUD available in their jail and completed this question (n = 982).

The logistic regression (Table 2) found that, typically, availability of all 3 types of MOUD was positively associated with urbanicity (B [SE], 1.45 [0.28] for large, 1.26 [0.27] for medium, 0.67 [0.30] for small metropolitan, and 0.63 [0.25] for micropolitan compared with noncore, or the most rural counties). Availability of methadone was similarly associated with urbanicity (B [SE], 1.38 [0.25] for large, 1.28 [0.24] for medium, 1.81 [0.26] for small metropolitan, and 1.05 [0.21] for micropolitan), but the association was less clear for buprenorphine and naltrexone.

**Table 2.** Logistic Regressions Identifying Factors Associated With Type of MOUD Available (N = 1243)^a^

Independent variable	All 3 types of MOUD available to at least some individuals		Buprenorphine available to at least some individuals		Methadone available to at least some individuals		Naltrexone available to at least some individuals	
	B (SE)	*P* value	B (SE)	*P* value	B (SE)	*P* value	B (SE)	*P* value
Urbanization								
Noncore	0 [Reference]	NA	0 [Reference]	NA	0 [Reference]	NA	0 [Reference]	NA
Large metropolitan	1.45 (0.28)	<.001	0.16 (0.26)	.53	1.38 (0.25)	<.001	1.10 (0.25)	<.001
Medium metropolitan	1.26 (0.27)	<.001	0.89 (0.25)	<.001	1.28 (0.24)	<.001	0.70 (0.24)	<.001
Small metropolitan	0.67 (0.30)	.03	−0.11 (0.27)	.67	1.81 (0.26)	<.001	0.32 (0.26)	.21
Micropolitan	0.63 (0.25)	.01	0.37 (0.20)	.07	1.05 (0.21)	<.001	1.06 (0.20)	<.001
Mean daily jail population								
0-25	0 [Reference]	NA	0 [Reference]	NA	0 [Reference]	NA	0 [Reference]	NA
26-50	0.49 (0.33)	.13	0.77 (0.28)	.01	0.21 (0.28)	.46	0.69 (0.29)	.02
51-100	−0.01 (0.31)	.97	0.24 (0.26)	.36	0.11 (0.27)	.68	1.29 (0.27)	<.001
101-200	0.30 (0.30)	.33	1.56 (0.28)	<.001	0.34 (0.26)	.20	0.86 (0.27)	<.001
>200	0.18 (0.29)	.53	1.17 (0.26)	<.001	0.48 (0.25)	.05	0.95 (0.26)	<.001
Jail has health professional available (either own staff or contracted) to administer MOUD	1.09 (0.35)	<.001	1.54 (0.24)	<.001	−0.51 (0.23)	.03	1.92 (0.30)	<.001
State expanded Medicaid (prior to 2022)	1.37 (0.21)	<.001	1.03 (0.16)	<.001	0.35 (0.15)	.02	0.80 (0.15)	<.001
Mean drive time to closest MOUD provider in county, min	0.01 (0.01)	.16	−0.02 (0.01)	.02	−0.01 (0.01)	.27	0.01 (0.01)	.28
2020 County opioid overdose mortality rate, deaths/100 000	0.02 (0.01)	<.001	0.001 (0.01)	.87	0.02 (0.01)	<.001	0.01 (0.01)	.19
Social Vulnerability Index Summary rank	−1.38 (0.33)	<.001	−1.61 (0.29)	<.001	−0.59 (0.27)	.03	−1.41 (0.28)	<.001

Abbreviations: MOUD, medications for opioid use disorder; NA, not applicable.

^a^ Data from 265 jails were weighted to be representative of 1243 jails nationwide.

Location in Medicaid expansion states was positively associated with offering all 3 medications to at least some detainees (B [SE], 1.37 [0.21]). Location in counties with higher overdose mortality rates was also positively associated with offering all 3 types of MOUD (mean [SD] rate, 29.6 [14.4] vs 25.6 [12.9] deaths per 100 000; B [SE], 0.02 [0.01]); however, when looking at each medication individually, only methadone was associated with county overdose mortality (mean [SD] rate, 28.9 [13.7] vs 24.8 [12.9] deaths per 100 000; B [SE], 0.02 [0.01]).

Higher county social vulnerability was negatively associated with availability of all 3 types of MOUD (B [SE], −1.38 [0.33]), buprenorphine (B [SE], −1.61 [0.29]), naltrexone (B [SE], −1.41 [0.28]), and methadone (B [SE], −0.59 [0.27]). Consistent with regulations regarding the administration of these medications, the availability of a health professional to administer MOUD was positively associated with offering all 3 types of MOUD (B [SE], 1.09 [0.35]), buprenorphine (B [SE], 1.54 [0.24]), and naltrexone (B [SE], 1.92 [0.30]), but not methadone (B [SE], −0.51 [0.23]).

Jails reported a variety of challenges, facilitators, and needs for administering MOUD (Table 3). The top challenges were policies and procedures (760 jails [78.7%] for buprenorphine; 699 jails [73.5%] for methadone) and logistical availability of the medication (611 jails [63.3%] for buprenorphine and 617 jails [64.9%] for methadone). Conversely, availability of staff (525 jails [70.0%] for buprenorphine; 243 jails [50.4%] for methadone) and jail policies and procedures (482 jails [64.2%] for buprenorphine; 243 jails [50.3%] for methadone) were the top facilitators. Overall, 667 jails (60.7%) indicated a need for additional medical staff. The most common technical assistance needs included staff education (670 jails [57.0%]) and connecting to community resources (614 jails [52.1%]). Similarly, the most common training needs were forming community linkage partnerships (508 jails [45.4%]) and partnering with community-based OTPs (373 jails [33.4%]). In addition, 410 jails (37.3%) indicated that they need help addressing stigma and negative attitudes toward MOUD.

**Table 3.** Challenges and Facilitators to Providing MOUD

Variable	Jails, No. (%) (N = 1243)
Buprenorphine challenges (n = 966)	
Policies and procedures (eg, security, diversion, DEA regulations)	760 (78.7)
Logistical accessibility of medication (eg, transportation, staff availability)	611 (63.3)
Community availability	180 (18.6)
Staff knowledge and attitudes	190 (19.7)
Patient knowledge, experiences, and attitudes	148 (15.3)
Methadone challenges (n = 951)	
Policies and procedures (eg, security, diversion, DEA regulations)	699 (73.5)
Logistical accessibility of medication (eg, transportation, staff availability)	617 (64.9)
Community availability	266 (28.0)
Staff knowledge and attitudes	166 (17.4)
Patient knowledge, experiences, and attitudes	100 (10.5)
Buprenorphine facilitators (n = 750)	
Availability of personnel to administer MOUD	525 (70.0)
State or county regulations	182 (24.3)
Jail policies and procedures	482 (64.2)
Funding	322 (43.0)
Long duration of stays in the jail	70 (9.3)
Availability in the community	332 (44.2)
Methadone facilitators (n = 483)	
Availability of personnel to administer MOUD	243 (50.4)
State or county regulations	100 (20.7)
Jail policies and procedures	243 (50.3)
Funding	111 (23.1)
Long duration of stays in the jail	46 (9.5)
Availability in the community	225 (46.5)
Resources needed to expand MOUD services within the jail (n = 1100)	
Additional medical staff	667 (60.7)
MOUD training for clinical staff within the jail	461 (41.9)
Same-day access to MOUD	454 (41.3)
Technical assistance for MOUD services	208 (28.0)
Help addressing stigma and negative attitudes toward MOUD	410 (37.3)
Information on how to become a licensed OTP	247 (22.5)
Information on MOUD and pregnant individuals	211 (19.2)
Technical assistance needs (n = 1176)	
Staff education about treatment of substance use disorders in general	670 (57.0)
Connecting people to community resources for substance use treatment	614 (52.1)
Screening tools	475 (40.4)
Counseling and shared decision-making tools for patients	284 (24.1)
Clinical practice guidelines on MOUD	273 (23.2)
Different types of MOUD and how to identify the best type for patient needs	269 (22.9)
Implementing treatment protocols	237 (20.1)
Training needs (n = 1119)	
Forming community linkage partnerships	508 (45.4)
Partnering with community-based OTPs to offer methadone to detainees	373 (33.4)
Memoranda of understanding to support reentry services	372 (33.3)
Activating or reactivating Medicaid	356 (31.9)
Administration, monitoring, and storage of medication	278 (24.9)
Pharmaceutical company trainers for injectable MOUD	162 (14.5)
Obtaining waivers to prescribe buprenorphine	115 (10.3)

Abbreviations: DEA, Drug Enforcement Agency; MOUD, medications for opioid use disorder; OTP, opioid treatment program.

## Discussion

Clinical guidance encourages jails to make all 3 types of MOUD available to all detainees with an OUD.^13,32^ However, in this survey study, we found that among the limited number of jails in which MOUD is available, only approximately one-quarter provided all 3 types (27.6%) and very few made them universally available (16.9%).

These findings indicate that several regulatory and logistical hurdles discourage jails from implementing MOUD. Jail policies and procedures, such as those related to security and diversion of medications, were cited as a top challenge for implementing both buprenorphine (78.7%) and methadone (73.5%). Recent research has shown that diversion of buprenorphine is both uncommon and preventable, and has suggested specific steps that can be readily implemented in jails.^33,34^ Regulations limiting the options for dispensing methadone are more challenging but not insurmountable.^35^ At least one-half of jails indicated that jail policies and procedures facilitated their use of MOUD.

Location in a Medicaid expansion state was associated with having all 3 types of MOUD available. Medicaid expansion has been associated with positive outcomes for this population,^36^ including increased coverage and receipt of MOUD among individuals recently released from correctional settings and among persons receiving services and supervision through problem-solving courts.^37,38,39^ Recent introduction of Medicaid Reentry Section 1115 Demonstration grants provides opportunity for states to seek approval to utilize Medicaid funds in support of MOUD during the critical reentry period.^23^ One-third of jails indicated a need for training in activating or reactivating Medicaid coverage as part of discharge planning. Recent legislation will prohibit canceling an individual’s Medicaid coverage if they become incarcerated; this should help facilitate care transitions and continuity of treatment at reentry.^40^ Future research should examine how Medicaid coverage impacts OUD treatment and outcomes for this population.

Additional solutions may be needed to ensure that sufficient and appropriate medical staff are available to administer MOUD within jails. The majority of jails (60.7%) indicated a need for additional medical staff. These staffing needs may be related to the general lack of medical staff and practitioners in jails across some regions of the US.^41^ Many jails (57.0%) cited the need for staff education about substance use disorder and effective treatment. Although less common, more than one-third of jails indicated that they need help addressing stigma and negative attitudes toward MOUD. Previous research has similarly found that stigma contributed to negative attitudes toward MOUD among criminal legal staff and that higher educational attainment was associated with more positive attitudes toward MOUD.^42^ Together, these findings suggest that providing training and education for staff may help improve implementation of jail-based MOUD services.

Universal access to MOUD for incarcerated populations remains elusive, and more research is needed to understand the barriers to universal availability of MOUD within jails. Among jails that have successfully implemented buprenorphine or methadone, only 53.0% and 23.6%, respectively, use them for both initiation of new patients and for continuation for those already receiving them in the community—that is, for all individuals with OUD. These jails represent success stories that warrant further examination and may inform implementation efforts elsewhere.

### Limitations

Our findings must be interpreted in light of several study limitations. Our part 2 survey achieved a response rate of 60.2%. Data from nonresponders might alter the descriptive statistics and jail-specific challenges reported in this article. Second, our use of a cross-sectional survey design limits our ability to draw causal inferences. Third, as with other self-report surveys, some participants might have underreported challenges they were facing in their facilities, either due to desirability bias or unawareness of the services currently offered. Fourth, some survey response options (eg, jail policies and procedures) were not defined and, therefore, were up to each jail’s interpretation. Fifth, we used mean drive times to assess local MOUD accessibility, which does not fully address access constraints and is dependent on the accuracy of the Substance Abuse and Mental Health Services Administration treatment locator. This approach is consistent with prior MOUD access research.^43,44^

## Conclusions

In this survey study of US jails, the findings highlight that even within jails offering MOUD, most do not provide the standard of care to all who need it. Although recent data suggest that MOUD is slowly becoming more available in jails,^3,21,27^ substantial efforts are needed to ensure that all detainees with OUD have access to their preferred treatment while incarcerated. A combination of policy, regulatory, financing, staffing, and educational solutions is urgently needed. Implementation strategies tested in ongoing Medicaid Reentry 1115 Demonstration projects and promotion of peer learning among jails that have successfully addressed these challenges offer the potential to increase the availability of these evidence-based treatments to a population that remains highly susceptible to overdose.

## Notes

Supplement.
Data Sharing Statement
