# Supplementary material for: Barriers to Universal Availability of Medications for Opioid Use Disorder in US Jails
Source: JAMA Netw Open. 2025 Apr 16;8(4):e255340. doi: 10.1001/jamanetworkopen.2025.5340 (PMC12004198; doi:10.1001/jamanetworkopen.2025.5340)
Supplement: Supplement. — Data Sharing Statement [file jamanetwopen-e255340-s001.pdf]

Data Sharing Statement

Flanagan Balawajder. Barriers to Universal Availability of Medications for Opioid Use Disorder in US Jails. *JAMA Netw Open*. Published April 16, 2025.
doi:10.1001/jamanetworkopen.2025.5340

Data
Data available: Yes
Data types: Deidentified participant data
How to access data: De-identified data from this study will eventually be made available to those interested with the use of a DUA through the JCOIN Data Commons.
When available: beginning date: 10-01-2025

Supporting Documents
Document types: None

Additional Information
Who can access the data: Researchers who enter into a DUA.
Types of analyses: The data are not yet available. This will be determined through a data disclosure review board.
Mechanisms of data availability: With a signed data access agreement.
